# Discovery of Novel Fetal Hemoglobin Inducers through Small Chemical Library Screening

**DOI:** 10.3390/ijms21197426

**Published:** 2020-10-08

**Authors:** Giulia Breveglieri, Salvatore Pacifico, Cristina Zuccato, Lucia Carmela Cosenza, Shaiq Sultan, Elisabetta D’Aversa, Roberto Gambari, Delia Preti, Claudio Trapella, Remo Guerrini, Monica Borgatti

**Affiliations:** 1 Department of Life Sciences and Biotechnology, University of Ferrara, Via Fossato di Mortara 74, 44121 Ferrara, Italy; giulia.breveglieri@unife.it (G.B.); cristina.zuccato@unife.it (C.Z.); csnlcr@unife.it (L.C.C.); shaiq.sultan@unife.it (S.S.); elisabetta.daversa@unife.it (E.D.); gam@unife.it (R.G.); 2 Department of Chemical and Pharmaceutical Sciences, University of Ferrara, Via Luigi Borsari 46, 44121 Ferrara, Italy; salvatore.pacifico@unife.it (S.P.); delia.preti@unife.it (D.P.); claudio.trapella@unife.it (C.T.); 3 Center of Biotechnology, University of Ferrara, Via Fossato di Mortara 64b, 44121 Ferrara, Italy

**Keywords:** hemoglobinopathies, *β*-thalassemia, sickle cell disease, fetal hemoglobin, chemical screening, compound library, cellular biosensors

## Abstract

The screening of chemical libraries based on cellular biosensors is a useful approach to identify new hits for novel therapeutic targets involved in rare genetic pathologies, such as *β*-thalassemia and sickle cell disease. In particular, pharmacologically mediated stimulation of human *γ*-globin gene expression, and increase of fetal hemoglobin (HbF) production, have been suggested as potential therapeutic strategies for these hemoglobinopathies. In this article, we screened a small chemical library, constituted of 150 compounds, using the cellular biosensor K562.GR, carrying enhanced green fluorescence protein (EGFP) and red fluorescence protein (RFP) genes under the control of the human *γ*-globin and *β*-globin gene promoters, respectively. Then the identified compounds were analyzed as HbF inducers on primary cell cultures, obtained from *β*-thalassemia patients, confirming their activity as HbF inducers, and suggesting these molecules as lead compounds for further chemical and biological investigations.

## 1. Introduction

The screening of chemical libraries is a useful approach for drug discovery and identification of new therapeutic molecules, by using in vitro assays based on enzymatic activity, fluorescence signaling, calcium or ionic efflux, and cellular biosensors. In this context, these tools can be important in identifying new hits for novel therapeutic targets involved in rare genetic pathologies, such as *β*-thalassemia and sickle cell disease (SCD).

The *β*-thalassemias are inherited hematological genetic disorders, caused by more than 300 mutations of the human *β*-globin gene, causing reduced or absent synthesis of adult *β*-globin, and a consequent excess of *α*-globins, which induces ineffective erythropoiesis and severe anemia [1]. The annual incidence at birth of symptomatic cases is estimated at 1/100,000 worldwide, with a great prevalence in the developing world [1]. The *β*-thalassemias can be grouped in different classes, depending on the *β*-globin gene mutations (*β*^0^ and *β^+^*-thalassemias) and on the transfusion dependency (transfusion-dependent and transfusion independent *β*-thalassemias). In the case of the most severe forms of *β*-thalassemia, regular blood transfusions and chelating therapies are the conventional therapeutic approaches [2,3,4,5]. While bone marrow transplantation is currently the only resolving treatment of the disease [6], several potentially curative strategies are under clinical validation, including gene therapy [7], gene editing [8], and treatment with novel agents potentiating hematopoiesis (i.e., luspatercept) [9].

SCD is a rare monogenic, hematological, and multi-organ disorder, associated with acute and chronic illness, and progressive organ damage [10]. The disease is due to a single point mutation (Glu6Val) on the HBB gene, causing polymerization of the mutant hemoglobin (Hb) S, and resulting in sickling of erythrocytes [11].

The clinical severity for these two hemoglobinopathies could be reduced by reactivating the expression of *γ*-globin, absent after birth. In this case, even a small increase in the percentage of fetal hemoglobin (HbF), replacing the deficient adult hemoglobin (HbA), could ameliorate the clinical picture, reducing anemia and transfusion burden in *β*-thalassemia, and inhibiting polymerization of sickle hemoglobin (HbS) in SCD [12,13,14]. This has been demonstrated by several findings, including the fact that hereditary persistence of fetal hemoglobin (HPFH) is associated with high HbF production in adults, causing clinically mild phenotypes, even in severe forms of *β*-thalassemia and SCD [15,16,17]. Accordingly, genome editing to create the HPFH genotype in erythroid precursor cells has been proposed as a useful approach for treating SCD and β-thalassemia [17]. In addition, several investigations have been instigated to identify effective HbF inducers [18,19,20,21,22].

Currently, hydroxyurea (HU) is the only FDA-approved therapeutic treatment for the induction of HbF in SCD patients [13,23]. With respect to *β*-thalassemia, in spite of its verified benefits in certain cases, demonstrated by the reach of a transfusion-independent phenotype in patients with *β*-thalassemia intermedia [24,25], specific approvals for *β*-thalassemia are lacking. On the other hand, adverse effects and potential toxicity in the case of long-term treatment are expected, and have been described for HU [26,27]. Furthermore, not all patients respond to HU treatment, or can become insensitive after repeated administrations [13,28]; therefore, the discovery and investigation of new HbF inducers is certainly needed.

In this work, we have screened a small chemical library constituted of 150 compounds, randomly selected, and exhibiting heterogeneous chemotypes, using our cellular biosensor K562.GR carrying enhanced green fluorescence protein (EGFP) and red fluorescence protein (RFP) genes under the control of the human *γ*-globin and *β*-globin gene promoters, respectively [29]. This system is suitable for identifying the induction ability on the transcription of *γ*-globin and *β*-globin genes in erythroid cells in an efficient and reproducible way [29,30]. This investigation resulted in the identification of a subset of compounds that were ultimately analyzed as Hb inducers on primary cell cultures obtained from *β*-thalassemia patients. This assay has been widely used to validate the HbF-inducing potential of molecules identified using large or middle-scale screening approaches [31,32,33].

## 2. Results

### 2.1. Screening of the Chemical Library Using the K562.GR Cellular Biosensor

In order to discover novel fetal hemoglobin (HbF) inducers from the collected chemical library, we performed a first screening based on the K562.GR reporter system. The clone K562.GR contains a reporter construct with EGFP (enhanced green fluorescent protein) and RFP (red fluorescent protein) genes under the control of the human γ-globin or *β*-globin gene promoters, respectively, and it has been validated as a high throughput screening (HTS) system in previous publications [29,30]. With this system, a potential HbF inducer can be easily identified by fluorescence-activated cell sorting (FACS) detection of the specific fluorescent protein, in response to the activation of the corresponding promoter. On the contrary, if the tested compound does not induce either the γ-globin or the *β*-globin promoter, no fluorescence is detected. Despite the fact that in this study we focused on HbF inducers, it should be underlined that the system would also allow the identification of transcription activators of the *β*-globin promoter, in the case RFP is increased instead of EGFP [29,30].

For each compound of the chemical library, four concentrations (10 μM, 1 μM, 100 nM, and 10 nM) were administered to K562.GR cells for 5 days, and then FACS analysis was performed.

**Figure 1:**
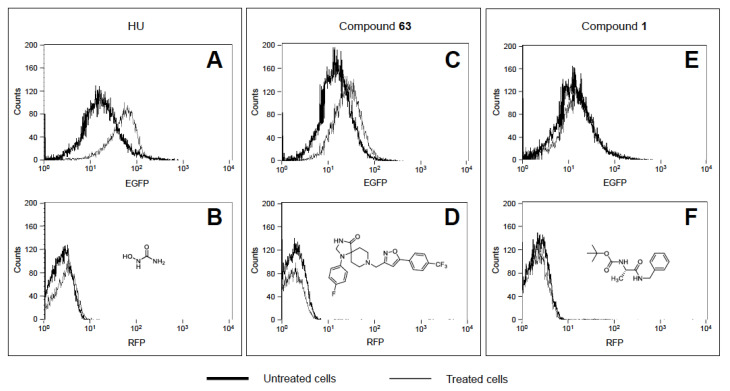
Representative examples of FACS analysis of K562.GR cells, untreated (bold solid lines) or treated (solid lines), with 150 µM HU (**A**,**B**), 10 µM compound 63 (**C**,**D**), and 10 µM compound **1** (**E**,**F**), respectively. The CheLiFe compounds were dissolved in DMSO. 30,000 cells were analyzed after five days of treatment, by detecting both enhanced green fluorescence protein (EGFP) (**A**,**C**,**E**) and RFP (**B**,**D**,**F**) fluorescence.

Figure 1 shows a representative example of the FACS analysis, obtained during the screening of the 150 compounds of the library, dissolved in dimethylsulfoxide (DMSO), plotting the curves generated by cells in the presence, and in the absence, of the treatment.

Hydroxyurea (HU) was selected as a positive control (Figure 1A), considering the fact that it has been firmly established to be able to induce γ-globin gene expression. For this reason, HU is used in clinical applications for *β*-thalassemia and SCA treatment [10]. As expected, when EGFP fluorescence, dependent on the activation of the γ-globin gene promoter, was measured (Figure 1A), a clear shift to the right of the curve for the HU treated cells (solid line), compared to the untreated cells (bold solid line), was obtained. On the contrary, no shift was observed when RFP fluorescence for *β*-globin gene promoter activation was analyzed (Figure 1B). After a qualitative analysis of the histograms obtained from all the compounds of the library, most of the molecules did not show detectable fluorescence increases, similarly to compound 1 (Figure 1E,F); the gaussian curves of the treated samples were perfectly superimposed on those of the untreated control, indicating the absence of an induction effect on both promoters. On the contrary, some of the analyzed compounds produced a shift to higher levels of EGFP fluorescence, similar to that found for compound 63 (Figure 1C,D). Interestingly, compound 63 (as HU) was also unable to induce RFP, confirming that its effects are restricted to the γ-globin gene promoter (associated to a clear increase of EGFP production).

In order to identify the compounds able to induce the γ-globin gene, we selected nine potentially active molecules, with values of median fold over control, for EGFP signal, greater than 1.35 in at least one of the four tested concentrations (Table 1 and Table S1 of Supplementary Materials).

Interestingly, while most of the compounds were active only at the highest concentration (10 µM), showing no induction activity at the other concentrations, some of them, such as 56, 58, and 59, also showed a relevant activity at low concentrations (1 µM). The 1.35 value was identified as a minimum cut-off in previous articles, where the same K562.GR cellular biosensor was validated using well-known fetal hemoglobin inducers, such as 4,4′,6-Trimethylangelicin (TMA) and HU [29,30,34].

In addition, the experiments shown in Figure 1, Table 1, and Table S1 (Supplementary Materials) were repeated using the same compounds dissolved in a methanol solution containing 5% DMSO. This was done considering that DMSO decreased the number of K562.GR cells (data not shown) when used as a negative control. Moreover, as reported in the literature, DMSO reduces the production of hemoglobin and represses the erythroid phenotype in K562 cells [35]. On the contrary, we demonstrated that methanol/DMSO does not affect the erythroid differentiation after treatment of immortalized K562.GR clones, showing no changes in relative EGFP (Figure S1A,C of Supplementary Materials) and RFP (Figure S1B,D of Supplementary Materials) fluorescence, detectable by FACS analysis.

Using methanol/DMSO, three independent experiments on K562.GR clones were performed (Table 2), in order to confirm the increase in EGFP levels, detected by FACS analysis in the previous experiments performed in DMSO (Table 1). The obtained median folds over control for EGFP analysis were greater than 1 and, in general, higher than the values measured during the first screening. In any case, we were able to confirm that the effects of the selected compounds were independent from the employed vehicle (DMSO versus methanol/DMSO).

### 2.2. Analysis of HbF Induction in a Human Erythroleukemic K562 Cell Line

The specificity of the induction activity of the nine compounds was determined by performing the same treatments on wild type K562 cells without the reporter construct. As reported in Table 3, the values of median fold over control were close to or less than 1, confirming for all nine compounds the absence of increased cellular fluorescence, and indicating that any fluorescence detection after the K562.GR treatment was presumably due to a specific induction of the γ-globin promoter, and not to an autofluorescence phenomenon of the employed compounds.

After this first screening, the effects of the selected compounds were investigated on cell growth, and on hemoglobin production by the K562 cell line. This cell line is a very useful experimental model system, since it synthesizes hemoglobin at very low levels when untreated, but significantly increases the expression of embryo-fetal globins after treatment with inducers of erythroid differentiation [36].

Table 4 reports the IC_50_ values (relative to the effects on cell proliferation) and the percentages of benzidine-positive cells calculated after 5 days of treatment. The analyzed compounds can be divided into three classes based on the relative IC_50_. The first one comprises 56, 58, and 59, showing the lowest values: 0.85 μM, 0.69 μM, and 0.10 μM, respectively. The second class includes compounds 6, 17, and 22, exhibiting intermediate values, between 1.2 and 5. Finally, for the other three molecules it was not possible to determine the IC_50_ value, since in our experimental conditions we did not detect significant antiproliferative effects at the maximum tested concentration (10 μM).

As far as the hemoglobin production analyzed using a benzidine test (Table 4), the same compounds showed an induction of effects on erythroid differentiation of K562 cells at different concentrations, and most of them in a dose-dependent manner. During this analysis, we noted that compounds with higher (>1 μM) or not determined IC_50_ (because it was greater than 10 μM) generally showed differentiation values dependent on the administered dose, even reaching quite high percentages of positive cells (for example, 15% for compound 6, 11% for compound 17, or 18% for compound 22, at 10 μM). In contrast, the three compounds with lower IC_50_ showed lower differentiation values, between 1 and 6%.

### 2.3. HbF Induction of Erythroid Precursors from β-Thalassemia Patients

The results obtained in K562 cell lines, prompted us to determine whether the nine molecules were able to induce the expression of γ-globin genes in primary cultures of erythroid precursor cells (ErPCs) obtained from *β*-thalassemia patients, evaluating the HbF production by HPLC. In total seven *β*-thalassemia patients were recruited, and their ErPCs isolated and treated as described elsewhere [37].

**Figure 2:**
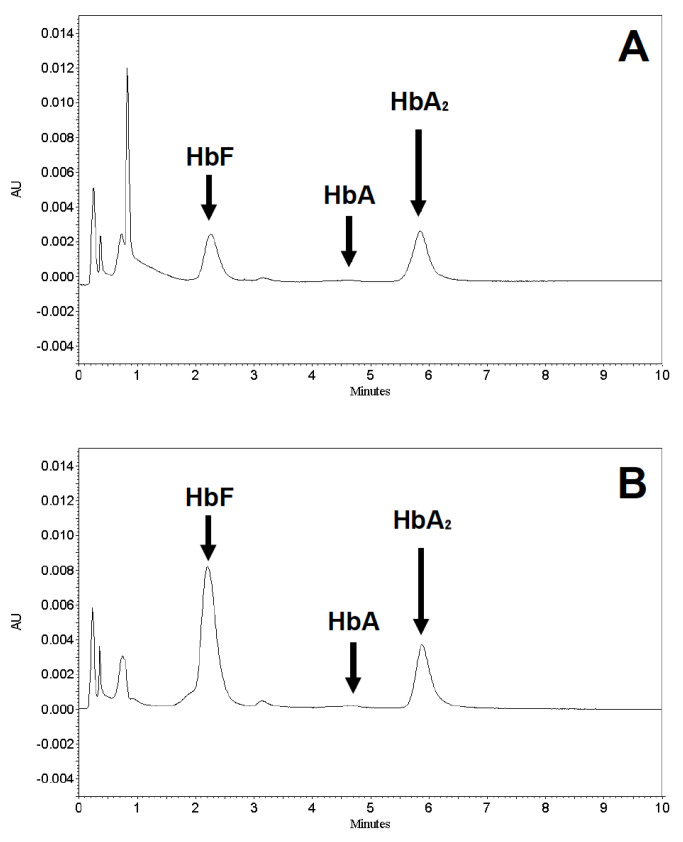
Representative example of HPLC analysis after erythroid precursor cells treatment. Chromatograms obtained from HPLC assay of erythroid precursor cells of patient 6 (a *β*-thalassemia patient), untreated (**A**) or after 5 days of treatment with 10 µM compound 63 (**B**). The positions of fetal (HbF) and adult (HbA ed HbA_2_) hemoglobin peaks are indicated by arrows.

A representative example of HPLC analysis after 5-day treatment of these isolated ErPCs is reported in Figure 2. The analysis of the Hb content of these cultures showed an increase of the HbF peak after the treatment with compound 63 at 10 µM (Figure 1B), relative to untreated cells (Figure 1A), while no relevant increase of the peaks corresponding to HbA and HbA_2_ was detected.

For each compound, we compared the HbF fold induction over untreated control values to those obtained after HU treatment (used as positive control) (Table 5).

In detail, compounds 6, 17, and 63 showed HbF fold induction values greater than that of HU in all treatments of erythroid precursor cells of different *β*-thalassemia patients, while the other compounds reported fold induction values greater than that of HU in one or two patient cultures. This result confirmed the erythroid differentiation ability of compounds 6 and 17 demonstrated in K562 cells (Table 4), suggesting their potential application as chemical inducers of HbF synthesis.

## 3. Discussion

Inherited hemoglobin disorders (such as sickle-cell disease and thalassemias) are among the most frequent monogenic diseases, originally characteristic of the tropics and subtropics, but now commonly distributed worldwide, mainly due to migration [38]. Hydroxyurea (HU) is currently the only FDA-approved drug for the induction of HbF in SCD patients, but it is effective in only 70% of patients, and a solution is required for the 30% non-responders [39], while regarding *β*-thalassemia patients, in spite of its sustained benefits for some untransfused patients with *β*-thalassemia intermedia [24,25], specific approvals for *β*-thalassemia are lacking. Therefore, HbF induction might be of great interest for the development of therapeutic protocols for these hemoglobinopathies [13,23,38]. In this context, chemical library screening using cellular biosensors can be considered a potential strategy to help the pharmaceutical development and identification of drugs for their therapeutic treatment. In this article we screened a small chemical library composed of 150 hits, and we identified two interesting lead molecules (compounds 6 and 17), confirming their HbF induction in different cellular models (cellular biosensor, immortalized erythroleukemic cell line, and primary cell cultures obtained from *β*-thalassemia patients) without cytotoxicity effects. In addition, compound 63 deserves consideration in future experiments, focusing on its mechanism for activity as a HbF inducer (see Figure 1 and Figure 2).

These data represent the first step of a process of drug discovery, that involves the identification of novel hits (considered in this study by the screening of a small library of randomly selected compounds), and the development of a program of medicinal chemistry, in order to produce analogues, to increase the affinity, selectivity, efficacy/potency, metabolic stability, and oral bioavailability of the identified lead compounds. In addition, these data represent the starting point for the future structure–activity relationship (SAR), in order to analyze the chemical structure/biological activity correlation, and identify the specific chemical group responsible for HbF increase in our cellular models. The SAR investigation will permit synthetizing new derivatives of the bioactive compounds 6, 17, and 63, modifying their chemical structure to improve their biological effect and potency with low toxicity.

Further experimental efforts on ErPCs isolated from *β*-thalassemia patients with different mutations of the *β*-globin gene, different HbF-related polymorphisms, and different starting levels of endogenously produced HbF are necessary to verify the therapeutic potential of the identified compounds. In addition, since SCD is expected to greatly benefit from production of anti-sickling HbF, ErPCs from SCD patients should be considered for experimental validation. Furthermore, combined treatments using compounds identified in the present study administered together with HbF-inducers employed in clinical trials (i.e., sirolimus, see clinical trials NCT03877809 and NCT04247750) might help to verify whether synergisms using different molecules can occur in inducing HbF. Finally, treatment with HbF inducers (including those described in this study) might also be combined with other strategies aimed at augmentation of HbF, for instance (a) inhibiting the activity of γ-globin gene transcriptional repressors (such as BCL11A, MYB, and ZBTB7A/LRF) [40,41,42,43], and (b) gene editing aimed at the genomic sequences negatively regulating γ-globin genes [44,45,46].

## 4. Materials and Methods

### 4.1. Chemical Library

The chemical library collects 150 chemical compounds developed at the University of Ferrara.

Each compound was dissolved in dimethylsulfoxide (DMSO) at a concentration of 10 mM, with a minimum degree of purity of 90%, evaluated by HPLC or mass analysis.

In the first screening of K562.GR clone, the compounds were tested in DMSO, while for the following experimental cultures they were diluted in methanol + 5% DMSO.

Analytical data (^1^HNMR spectra and found (M+H) + ESI mass) of the reference compounds of the manuscript are reported in Table S2, Supplementary Materials.

### 4.2. Cell Lines and Culture Conditions

The human erythroid leukemia K562 cells [47] and the derived cellular biosensor K562.GR [29,30] were cultured in RPMI 1640 medium (Sigma Aldrich, St. Louis, MO, USA) containing 10% fetal bovine serum (FBS; Biowest, Nuaillé, France) with addition of 100 units/mL penicillin and 100 μg/mL streptomycin (Pen-Strep; Lonza Group, Basel, Switzerland).

Experimental cultures were carried out by adding the appropriate drug concentrations to the cells usually seeded at 8000 cells/mL in 24-well plates. Cell growth was determined as cell number per ml using a Z2 Coulter (Beckman Coulter, Brea, CA, USA). The number of K562 cells containing hemoglobin was assayed by benzidine staining as elsewhere described [37].

### 4.3. FACS Analysis

For the determination of fluorescence intensity of treated K562 and K562.GR clones, BD FACSCanto™ II instrument and BD FACSDiva™ Software (Becton Dickinson, Franklin Lakes, NJ, USA) were used. After a PBS washing step, 30,000 cells were analyzed by the fl1 channel to detect green fluorescence. The results were expressed as median fold over control, that is the ratio between the median of fluorescence intensity values obtained by cells in the presence, and in the absence, of treatment.

### 4.4. Erythroid Precursor Cultures Obtained from Blood of Thalassemia Patients

Blood samples were collected from the Thalassemic Day Hospital (DHT) of the University Hospital “Sant’Anna” (Cona, Ferrara, Italy). Written informed consent was obtained from each patient, and peripheral blood samples were collected just before the blood transfusion. The primary cell cultures were obtained using a two-phase liquid culture procedure, as previously described [37,48]. Briefly, mononuclear cells were isolated from blood by density gradient centrifugation, and cultured in *α*-MEM (Sigma Aldrich, St. Louis, MO, USA), 10% FBS, 1 µg/mL cyclosporine A (Sigma Aldrich, St. Louis, MO, USA), 10% conditioned medium from the 5637 bladder carcinoma cell line and 10 ng/mL stem cell factor (SCF; Life Technologies, Carlsbad, CA, USA). After seven days incubation at 37 °C and in an atmosphere of 5% CO_2_, the non-adherent cells were harvested, washed, and cultured in fresh medium composed of *α*-MEM, 30% FBS, 1% deionized bovine serum albumin (BSA; Sigma Aldrich, St. Louis, MO, USA), 10^−5^ M *β*-mercaptoethanol (Sigma Aldrich, St. Louis, MO, USA), 2 mM L-glutamine (Sigma Aldrich, St. Louis, MO, USA), 10^−6^ M dexamethasone (Sigma Aldrich, St. Louis, MO, USA), 1 U/mL human recombinant erythropoietin (Tebu-bio, Magenta, MI, Italy), and 10 ng/mL SCF. After seven days of incubation, the cells were seeded at 1 × 10^6^/well in a 12-well plate, and treated with compounds for five days before the HPLC analysis to determine the proportion of HbF (as percentage of total Hb). In particular, a Beckman Coulter instrument System Gold 126 Solvent Module-166 Detector was used, in combination with a Syncropak CCM 103/25 column eluting the samples in a solvent gradient using aqueous sodium acetate-BisTris-KCN buffers, as previously reported [37,48]. The calculated percentages were used to evaluate HbF induction after the treatment, taking into consideration the starting hemoglobin levels of each individual patient, by applying the following algorithm: % induction = ((% HbF after treatment—% HbF before treatment)/(100—% HbF before treatment)) × 100.

## Figures and Tables

**Table 1:** Values of median fold over control for EGFP signals, expressed as mean value ± standard deviation, obtained from three different treatments of K562.GR cells with each compound at 10 nM, 100 nM, 1 μM and 10 μM concentration. The CheLiFe compounds were dissolved in DMSO. Compound 56 resulted toxic at 10 μM (−). * Structure non disclosable for patenting reasons.

Compound	Structure	10 nM	100 nM	1 µM	10 µM
6	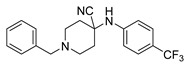	1.15	1.05	1.00	1.57
17	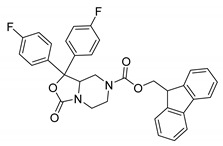	0.90	0.94	0.99	1.48
22	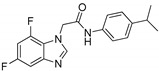	0.94	0.92	1.02	1.53
56	ND *	1.02	1.05	1.59	-
57	ND *	1.10	1.00	1.07	1.36
58	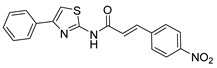	0.99	1.13	1.55	1.62
59	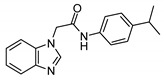	1.03	1.20	2.00	2.10
62	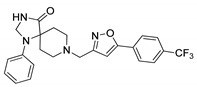	1.01	0.96	0.98	1.35
63	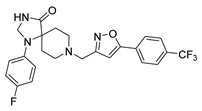	1.02	1.01	1.04	1.78

* Structure non disclosable for patenting reasons.

**Table 2:** Values of median fold over control for EGFP signals, expressed as mean value ± standard deviation, obtained from three different treatments of K562.GR cells, with each compound at 10 nM, 100 nM, 1 µM, and 10 µM concentration. The CheLiFe compounds were dissolved in methanol +5% DMSO. Compounds 56 and 59 resulted toxic at 10 µM (−).

Compd	10 nM	100 nM	1 µM	10 µM
6	1.09 ± 0.08	1.11 ± 0.07	1.14 ± 0.06	1.47 ± 0.27
17	1.13 ± 0.07	1.17 ± 0.05	1.18 ± 0.04	1.42 ± 0.13
22	1.11 ± 0.08	1.19 ± 0.06	1.29 ± 0.05	1.89 ± 0.27
56	1.11 ± 0.05	1.19 ± 0.12	1.47 ± 0.20	-
57	1.13 ± 0.12	1.13 ± 0.09	1.21 ± 0.10	1.60 ± 0.30
58	1.13 ± 0.07	1.20 ± 0.11	1.53 ± 0.31	1.54 ± 0.20
59	1.14 ± 0.06	1.38 ± 0.24	1.77 ± 0.15	-
62	1.15 ± 0.11	1.18 ± 0.05	1.24 ± 0.09	1.61 ± 0.28
63	1.14 ± 0.09	1.17 ± 0.07	1.20 ± 0.08	1.63 ± 0.28

**Table 3:** Values of median fold over control for EGFP signals, expressed as mean value ± standard deviation, obtained from three different treatments of K562 cells, with each compound at 10 nM, 100 nM, 1 µM, and 10 µM concentration. The CheLiFe compounds were dissolved in methanol +5% DMSO. Compounds 56 and 59 resulted toxic at 10 µM.

Compound	10 nM	100 nM	1 µM	10 µM
6	0.76 ± 0.17	0.67 ± 0.15	0.72 ± 0.17	0.57 ± 0.02
17	0.70 ± 0.19	0.62 ± 0.16	0.58 ± 0.12	0.50 ± 0.03
22	0.70 ± 0.23	0.62 ± 0.15	0.61 ± 0.13	0.49 ± 0.04
56	0.75 ± 0.15	0.69 ± 0.14	0.72 ± 0.24	-
57	0.75 ± 0.25	0.63 ± 0.22	0.65 ± 0.21	0.63 ± 0.22
58	0.73 ± 0.18	0.67 ± 0.18	1.07 ± 0.43	0.79 ± 0.24
59	0.76 ± 0.20	0.66 ± 0.18	0.83 ± 0.43	-
62	0.69 ± 0.25	0.60 ± 0.24	0.61 ± 0.22	0.60 ± 0.23
63	0.74 ± 0.27	0.63 ± 0.21	0.61 ± 0.23	0.58 ± 0.19

**Table 4:** Effects of the nine compounds from the CheLiFe library, selected after screening with the K562.GR reporter system, on K562 cell proliferation and differentiation. The IC_50_ value, determined, when possible, by counting K562 cells after 5 days from administration, is reported for each compound, together with the percentage of benzidine-positive cells after treatment with different concentrations of compounds for 7 days.

Compound	IC_50_ (µM)	Benzidine Assay	
		Concentration	Benzidine-Positive Cells (%)
6	5.00	1 µM	4
		5 µM	9
		10 µM	15
17	3.60	1 µM	6
		5 µM	10
		10 µM	11
22	1.20	1 µM	4
		5 µM	7
		10 µM	18
56	0.85	0.5 µM	1
		1 µM	2
		5 µM	1
57	>10.00	1 µM	5
		5 µM	6
		10 µM	8
58	0.69	0.5 µM	6
		1 µM	5
		5 µM	3
59	0.10	0.5 µM	2
		1 µM	3
		5 µM	2
62	>10.00	1 µM	3
		5 µM	4
		10 µM	8
63	>10.00	1 µM	2
		5 µM	3
		10 µM	6

**Table 5:**
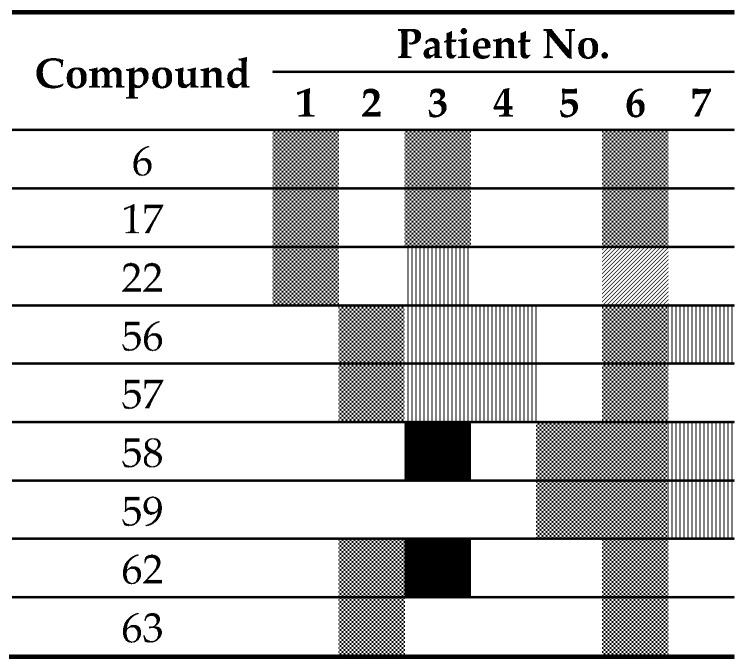
Graphic representation of fold induction over control values for HbF production, obtained after treatment of erythroid precursor cells of seven different *β*-thalassemic patients with the nine compounds under investigation.

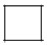
 not treated; 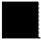 not analyzable; 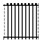 fold induction <50% HU; 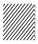 50% HU <fold induction <HU; 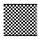 fold induction >HU.

## Supplementary Materials

The following are available online at https://www.mdpi.com/1422-0067/21/19/7426/s1, Figure S1: Representative examples of FACS analysis of K562.GR cells, Table S1: Values of median fold over control obtained after treatment of K562.GR cells, Table S2: Analytical data (1HNMR spectra and found [M+H]+ ESI mass) of the reference compounds of the manuscript.
