# Supplementary material for: Discovery of Novel Fetal Hemoglobin Inducers through Small Chemical Library Screening
Source: Int J Mol Sci. 2020 Oct 8;21(19):7426. doi: 10.3390/ijms21197426 (PMC7582302; doi:10.3390/ijms21197426)
Supplement: Supplementary file 1 [file ijms-21-07426-s001.zip › SUPPLEMENTARY MATERIALS/Figure 1S.pdf]

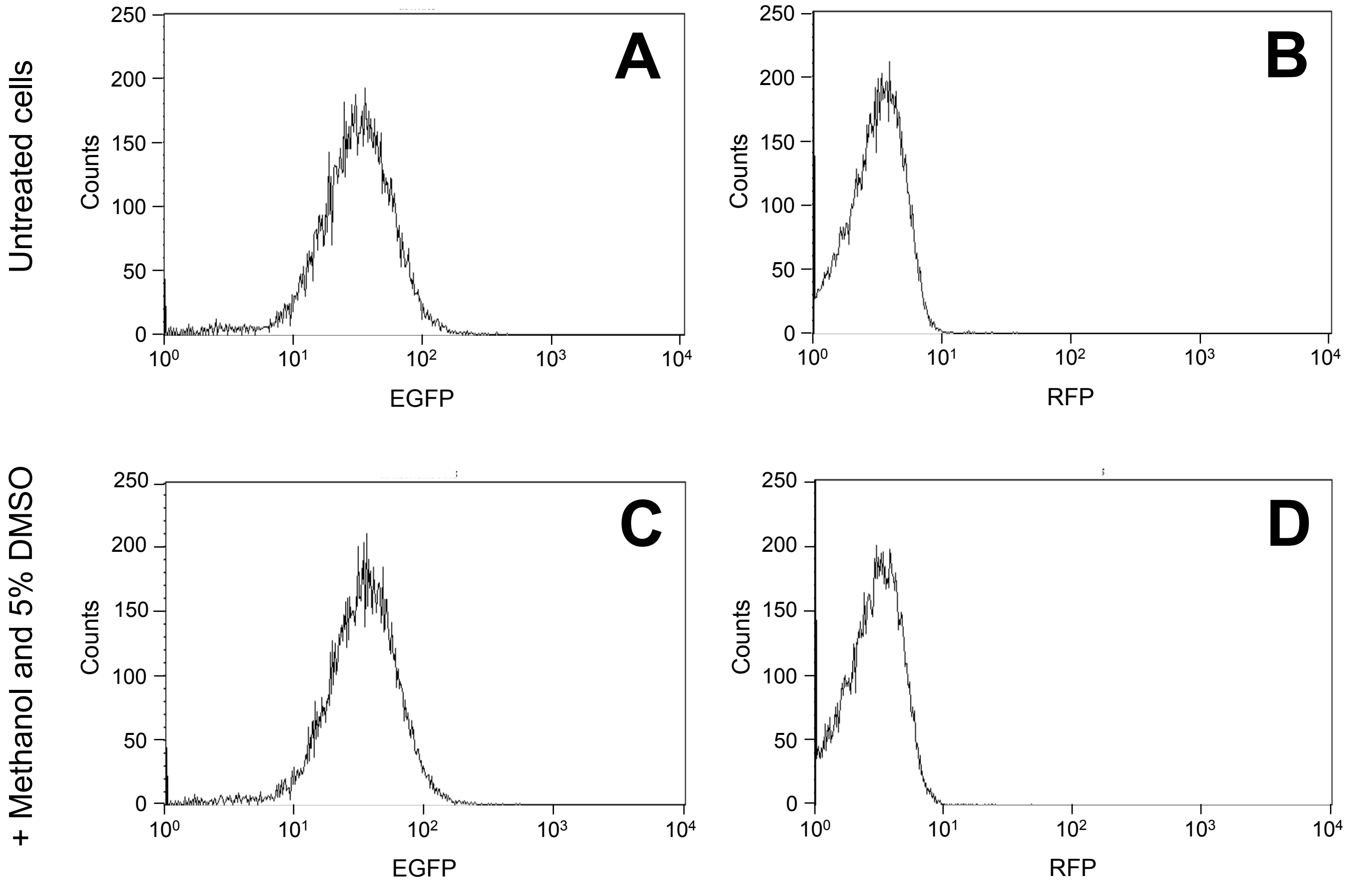

Figure 1S. Representative examples of FACS analysis of K562.GR cells, untreated (A, B) or treated with a methanol solution containing 5% DMSO (C, D). 30.000 cells were analyzed after five days of treatment, by detecting both EGFP (A, C) and RFP (B, D) fluorescence.
