# Supplementary material for: Discovery of Novel Fetal Hemoglobin Inducers through Small Chemical Library Screening
Source: Int J Mol Sci. 2020 Oct 8;21(19):7426. doi: 10.3390/ijms21197426 (PMC7582302; doi:10.3390/ijms21197426)
Supplement: Supplementary file 1 [file ijms-21-07426-s001.zip › SUPPLEMENTARY MATERIALS/Table IIs.docx]

| **Compd** | **Structures** | **NMR** | **[M+H]^+^** |
| --- | --- | --- | --- |
| **6** |  | ^1^HNMR (200 MHz, CDCl_3_), δ (ppm): 7.48 (d, J = 7.5 Hz, 2H), 7.35-7.24 (m, 5H), 6.88 (d, J = 7.5 Hz, 2H), 4.05 (s, 1H), 3.59 (s, 2H), 2.93-278 (m, 2H), 2.56-2.35 (m, 4H), 2.1-1.85 (m, 2H). | 359.93 |
| **17** |  | ^1^H NMR (200 MHz, DMSO-d6), δ (ppm): 7.94-7.84 (m, 2H), 7.62-7.18 (m, 14H), 4.65-4.23 (m, 4H), 3.95-3.85 (m, 1H), 3.87-3.77 (m, 1H), 3.05-2.85 (m, 3H), 2.31-2.16 (m, 1H). | 553.57 |
| **22** |  | ^1^H NMR (200 MHz, DMSO-d6), δ (ppm): 8.31 (s, 1H), 7.46 (d, J = 8 Hz, 2H), 7.40 (m, 1H), 7.20 (d, J = 8 Hz, 2H), 7.17-7.14 (m, 1H), 5.23 (s, 2H), 2.83 (hept, J = 7 Hz, 1H), 1.16 (d, J = 7 Hz, 6H). | 330.27 |
| **58** |  | ^1^HNMR (200 MHz, CDCl_3_), δ (ppm): 8.27-8.20 (m, 2H), 7.89-7.78 (m, 3H), 7.65 (d, J = 7.5 Hz, 1H), 7.57-7.40 (m, 5H), 6.60 (d, J = 15.0 Hz, 1H), 6.40 (d, J = 15.0 Hz, 1H). | 352.36 |
| **59** |  | ^1^HNMR (200 MHz, CDCl_3_) δ (ppm): 8.61 (s, 1H), 8.20 (s, 1H), 7.76-7.71 (m, 1H), 7.52-7.46 (m, 1H), 7.41- 7.30 (m, 3H), 7.08 (d, J = 8 Hz, 2H), 5.07 (s, 2H), 3.74 (s, 1H), 2.81 (hept, J = 6.2 Hz, 1H), 1.16 (d, J = 7 Hz, 6H). | 294.44 |
| **62** |  | ^1^HNMR (400 MHz, DMSO-d6) δ (ppm): 8.67 (s, 1H), 8.12 (d, J = 8 Hz, 2H), 7.90 (d, J = 8 Hz, 2H), 7.29-7.22 (m, 3H), 6.86 (d, J = 7.5 Hz, 2H), 6.77-6.69 (m, 1H), 4.57 (s, 2H), 3.68 (s, 2H), 2.86-2.54 (m, 4H), 2.57-2.48 (m, 2H), 1.62-1.57 (m, 2H). | 457.40 |
| **63** |  | ^1^HNMR (400 MHz, DMSO-d6) δ (ppm): 8.16 (s, 1H), 8.10 (d, J = 8 Hz, 2H), 7.88 (d, J = 8 Hz, 2H), 7.24 (s, 1H), 7.14-7.07 (m, 2H), 6.94-6.88 (m, 2H), 4.53 (s, 2H), 3.64 (s, 2H), 2.82-2.69 (m, 4H), 2.32-1.99 (m, 2H), 1.64-1.59 (m, 2H). | 475.37 |

**Table IIs.** Analytical data (^1^HNMR spectra and found [M+H]^+^ ESI mass) of the reference compounds of the manuscript. Compound **58** has been recently reported by Sivakumar et al. (European Journal of Biomedical and Pharmaceutical Sciences, 2018, 5, 246-256) and evaluated for antimicrobial activity. To the best of our knowledge, none of the remaining compounds has been reported to date in scientific literature. These molecules are intermediates or final compounds isolated and characterized during different organic/medicinal chemistry research programs and their synthesis may be the subject of future publications relating to the original projects.
